# Long-term CIN3+ risk in women with abnormal cytology; role of hrHPV testing

**DOI:** 10.1038/bjc.2012.5

**Published:** 2012-02-14

**Authors:** M Kocken, J Berkhof, F J van Kemenade, J A Louwers, A Zaal, M A E Nobbenhuis, G Kenter, P J F Snijders, C J L M Meijer, T J M Helmerhorst

**Affiliations:** 1 Department of Obstetrics and Gynaecology, Erasmus MC University Medical Centre, PO Box 2040, 3000 CA Rotterdam, The Netherlands; 2 Department of Pathology, VU University Medical Centre, PO Box 7057, 1007 MB Amsterdam, The Netherlands; 3 Department of Epidemiology and Biostatistics, VU University Medical Centre, PO Box 7057, 1007 MB Amsterdam, The Netherlands; 4 University Medical Centre Utrecht, Division of Woman and Baby, Gynaecological Oncology, PO Box 85500, 3508 GA Utrecht, The Netherlands; 5 Department of Gynaecological Oncology, The Royal Marsden Hospital, Fulham Road, SW3 6JJ London, UK; 6 Department of Obstetrics and Gynaecology, Centre Gynaecological Oncology Amsterdam (AMC, NKI-AVL, VUmc), VU University Medical Centre, PO Box 7057, 1007 MB Amsterdam, The Netherlands

**Keywords:** cervical cytology, human papillomavirus, cervical intraepithelial neoplasia, triage, screening

## Abstract

**Background:**

Many studies have examined the short-term value of high-risk human papillomavirus (hrHPV) testing in predicting cumulative risk of cervical intraepithelial neoplasia grade 3 or cancer (CIN3+). This study focuses on long-term CIN3+ risk after initial wait and see policy.

**Methods:**

A total of 342 women with abnormal cytology of borderline/mild dyskaryosis (BMD) or worse (>BMD), included between 1990 and 1992, were followed-up by cytology and hrHPV testing until 1996 and monitored by cytology thereafter. Primary endpoint was cumulative CIN3+ risk by December 2009.

**Results:**

Women with BMD had a 5-year CIN3+ risk of 22.5% (95% confidence interval (CI) 17.0–29.1) and of 0.7% (0.1–4.5) in the subsequent 5 years. High-risk human papillomavirus-negative women with BMD had a 5-year risk of <0.01% (95% CI 0.0–5.1) and of <0.01% (0.0–5.7) in the following 5 years, while for hrHPV-positive women these risks were 37.5% (29.0–46.9) and 1.6% (0.2–9.5), respectively. Women with >BMD had a 5-year risk of 45.1% (36.4–54.1) and of 3.5% (0.9–12.2) in the subsequent 5 years. High-risk human papillomavirus-negative women with >BMD had a 5-year risk of 7.3% (2.0–23.6) and hrHPV-positive women of 56.6% (46.4–66.3).

**Conclusion:**

Women with BMD have an elevated CIN3+ risk for 5 years only; afterwards their risk is similar to the general population. High-risk human papillomavirus-negative women with BMD may return to regular screening directly. All other women with ⩾BMD should be referred for additional testing and/or colposcopy.

The incidence of cervical cancer has been lowered by the implementation of population-based screening programmes in which women are screened by cytological testing (Gustafsson *et al*, 1997; van Ballegooijen and Hermens, 2000; Castle *et al*, 2007). However, the sensitivity of cytological testing for cervical intraepithelial neoplasia grade 3 or cervical cancer (CIN3+) is moderate and compensated for by repetitive screening (Manos *et al*, 1999; Arbyn *et al*, 2006). In the Netherlands, an abnormal cytological test result is detected in ∼2–4% of all screened women (van Ballegooijen and Hermens, 2000; Bulk *et al*, 2004; Bulkmans *et al*, 2004). In most developed countries, women with minor cellular abnormalities of borderline and mild dyskaryosis (BMD) will be followed by cytology, and will be referred for colposcopy if the smear remains abnormal (van Ballegooijen and Hermens, 2000; Bulkmans *et al*, 2004, 2007). Women with moderate and severe abnormalities (>BMD) are referred for colposcopy (van Ballegooijen and Hermens, 2000; Bulkmans *et al*, 2004). However, a substantial proportion of women with abnormal cytology will regress or do not harbour clinically meaningful cervical disease and will therefore be unnecessarily retested or referred.

Infection with a high-risk type of human papillomavirus (hrHPV) is the causative agent in cervical cancer (Walboomers *et al*, 1999; Bosch *et al*, 2002). Molecular testing for hrHPV has a higher sensitivity than cytology to detect CIN3+ (Manos *et al*, 1999; Cuzick *et al*, 2003, 2008; Arbyn *et al*, 2004, 2006; Bulkmans *et al*, 2007; Mayrand *et al*, 2007). In women with abnormal cytology, studies focus on the additional value of hrHPV in triaging women with equivocal or mildly abnormal cytological test results in order to increase efficiency of patient management (i.e., referral for colposcopy) and to identify women with an increased risk for high-grade CIN. As most of these studies had a restricted follow-up of at maximum 6 years, little is known on risk profiles with longer periods of follow-up and the effect of hrHPV testing in those situations (Manos *et al*, 1999; Nobbenhuis *et al*, 1999; Cuzick *et al*, 2003; Arbyn *et al*, 2004; Bais *et al*, 2005; Bulk *et al*, 2007; Safaeian *et al*, 2007; Castle *et al*, 2009; Rijkaart *et al*, 2009; Cotton *et al*, 2010; Katki *et al*, 2011; Kelly *et al*, 2011; Kitchener *et al*, 2011; Levi *et al*, 2011). Only few studies have reported about a follow-up period of over 10 years (Sherman *et al*, 2003; Khan *et al*, 2005; Schiffman *et al*, 2011).

In this study we followed a group of women who were diagnosed with an abnormal cytology result of ⩾BMD for a maximum of 19 years and evaluated their long-term cumulative risk of developing CIN3+. Also the value of hrHPV testing for risk assessment was established as well as the duration of follow-up needed for women with dyskaryosis.

## Materials and methods

### Study population

**Figure 1:**
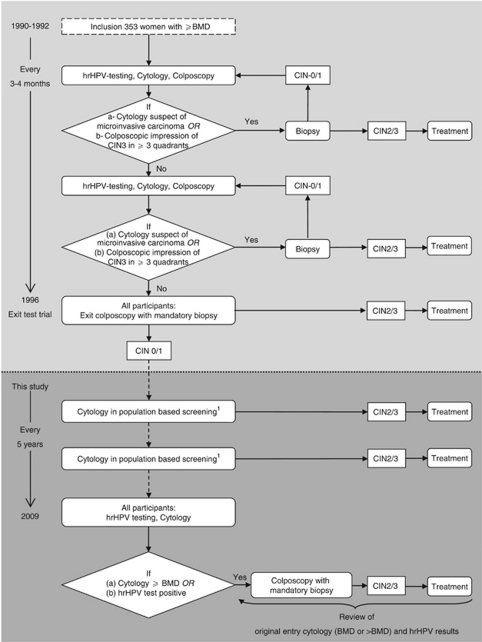
Flowchart of follow-up procedures. ^1^ Referral for colposcopy when once a cytology result of >BMD or when twice a result of BMD is detected. Abbreviations: BMD, borderline or mild dyskaryosis; hrHPV, high-risk human papillomavirus; CIN, cervical intraepithelial neoplasia.

For this cohort study we followed-up women who had participated in a previous study that studied the association between the presence of hrHPV and the development of high-grade cervical lesions (Nobbenhuis *et al*, 1999). Detailed methods of recruitment and follow-up until 33 months after intake have been published previously (Nobbenhuis *et al*, 1999, 2001). In short, between June 1990 and December 1992, 353 women were referred to the colposcopic outpatient clinic (VU University medical centre, Amsterdam, The Netherlands) with an abnormal cervical cytology result of mild, moderate or severe dyskaryosis. Until December 1996 each participant had been monitored for cervical disease every 3–4 months by testing for hrHPV, cytology, and colposcopy. Three expert colposcopists assessed serial colpophotographs and gave a consensus impression of the lesion. Only when they suspected a CIN3 lesion covering three or more cervical quadrants, or when a cervical smear result suspect of cervical cancer was found, a biopsy had been taken. At the end of the study in December 1996, all women had a colposcopic examination with mandatory biopsy (median 36 months, range 1–75). Women identified with high-grade disease (CIN2+) were treated according to Dutch guidelines. In Figure 1 follow-up procedures are depicted in a flowchart.

### Procedures

Cytology results were originally reported using a classification that predated the currently used classification; therefore, all cytological referral slides were retrieved from the archives for blind review by an expert gynaeco-pathologist (FvK). Dotted slides were scored and dichotomised into (⩽) BMD, or >BMD. Women of whom no referral slide could be retrieved were excluded from this study.

Between December 1996 and December 2009 all women were monitored by cytological population-based screening once every 5 years. Interim-colposcopies were performed according to the national guidelines (NVOG, 2004). To complete the data obtained from routine screening, we invited all women to visit the outpatient clinic (VU University medical centre) for additional cytology and hrHPV testing during 2009 (Figure 1). If travel distance was a limitation to participate, women were offered the possibility of performing a hrHPV test at home by self-sampling. These test results are similar to those acquired by a physician (Bais *et al*, 2007; Petignat *et al*, 2007). Women who had had a hysterectomy were censored at the date of hysterectomy.

Two cervical specimens were obtained from women who visited the outpatient clinic (Cervex-brush, Rovers Medical Devices, Oss, The Netherlands). The first specimen was collected in a liquid-based cytology medium (Surepath, Tripath Imaging, Burlington NC, USA), cytologically examined, and classified according to the CISOE-A classification, which is easily translatable into the Bethesda 2001 classification (Bulk *et al*, 2004). The second specimen was stored in Universal Collection Medium (Qiagen Corporation, Gaithersburg, MD, USA) for hrHPV testing. Women who self-sampled returned their cervicovaginal specimen for hrHPV testing by mail. All hrHPV samples were tested with the clinically validated GP5+/6+ PCR with enzyme-immunoassay read-out using a cocktail probe for 14 hrHPV types (16, 18, 31, 33, 35, 39, 45, 51, 52, 56, 58, 59, 66 and 68), according to established protocols (van den Brule *et al*, 2002; Snijders *et al*, 2005). The PCR products of hrHPV-positive women were subsequently genotyped by reverse line blot hybridisation. Samples that were negative for any specific probe in this reverse hybridisation assay were considered positive for uncharacterised subtypes or variants (HPV X).

A standard colposcopic assessment was performed when a cytological test was abnormal at the threshold of borderline dyskaryosis, or when the hrHPV test was positive (Figure 1). Biopsies were taken of all suspect lesions. Histological specimens were graded as CIN0 (no dysplasia), CIN1, CIN2, CIN3, adenocarcinoma *in situ* (AIS) or invasive cancer (Wright, 1995) and classified according to the highest abnormality found in biopsy or treatment specimen. Women who developed CIN2+ were treated according to present guidelines but were censored at the time of treatment.

In December 2009, the hospital database and the Netherlands nationwide network and registry of histopathology and cytopathology (PALGA; Bunnik, The Netherlands) were reviewed for all women, irrespective of attendance, to ascertain details of any additional relevant events and procedures. Ethical approval was obtained from the Ethics Board of the VU University medical centre. All women who attended the outpatient clinic or participated by self-sampling provided additional signed informed consent. The study is registered in the Dutch trial register (NTR1470).

### Statistical methods

In order to report long-term CIN risks in women with dyskaryosis, this study was designed as a follow-up of an observational cohort (Nobbenhuis *et al*, 1999).

As the original study was designed such that no interference with natural history would occur; a biopsy had only been taken when a colposcopic impression of CIN3 covering three or more cervical quadrants was present, or when a cytology result was suspect of cancer. As a consequence, the exact time at which CIN3+ lesions had developed was difficult to assess. We have calculated the 5-year, 10-year and overall risks until detection of CIN3+ using different approaches. In the first approach, we equalled the event time to the time of the first abnormal cytological result of moderate dyskaryosis or worse. In the second approach, the event time was equalled to the time of histological diagnosis. As the difference between the risks of these approaches were minimal (data not shown), we applied the second approach in further analyses. In women without an event, data were right-censored at the date of the last registered test.

The primary endpoint was the cumulative risk of CIN3+. We repeated the calculations with CIN2+ as secondary endpoint because treatment of CIN2 is common practice in most western countries. Both CIN3+ and CIN2+ included cases of AIS, adenocarcinoma (AC) and squamous cell carcinoma (SCC).

The cumulative CIN3+ risk was estimated by Kaplan–Meier analysis for the total group as well as for subgroups of different cytological and hrHPV test results at time of referral. In addition we repeated the calculations after dichotomising in younger (<30 years) and older (⩾30 years) women. Differences in cumulative risk curves between subgroups were assessed by log-rank tests.

For women who did not develop high-grade CIN within 6 months after inclusion, we reset the time at 6 months to 0 to estimate the value of retesting with cytology, hrHPV or both after 6 months and the risk of persistent hrHPV infection (log-rank tests). For women who had not developed high-grade CIN at 5 years after inclusion, time was reset from 5 years to 0 to estimate the CIN3+ risk from 5 years onwards.

By Cox regression we calculated CIN hazard ratios and 95% confidence intervals (CIs) to compare different test result combinations. Overall cumulative risks were calculated for different hrHPV genotypes to determine whether genotyping has additional value in the follow-up of women with abnormal cytology, focusing on HPV16. All calculations were performed using SPSS (Version 17.0, SPSS Inc, Chicago, IL, USA). All tests were two-sided and the level of significance was set at 0.05.

## Results

Of the original 353 women, 11 (3.1%) were excluded as no referral slide could be retrieved for review. For the remaining 342 women (median age 31 years, range 17–54) maximum follow-up depended on accrual date and ranged from 17.0 to 19.5 years. The total number of women years in our study was 3152. Overall censoring percentages were 13.2% (45 out of 342) at 5 years, 21.6% (74 out of 342) at 10 years and 36.5% (125 out of 342) at 15 years after detection of an abnormal cytological test result. During follow-up 4 women died of unrelated disease, 6 moved abroad, and 23 had a hysterectomy. None of the women had received prophylactic hrHPV vaccination.

**Figure 2:**
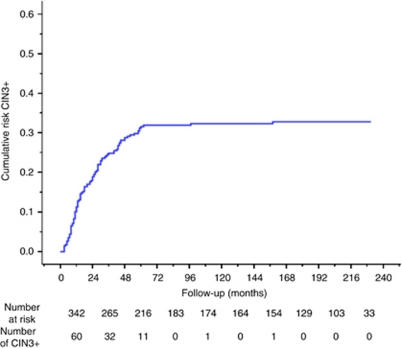
Cumulative risk curve of CIN3+ in 342 women with abnormal cytology (mild to severe dyskaryosis) at baseline. Abbreviation: CIN3+, Cervical intraepithelial neoplasia grade 3 or cancer.

During follow-up 105 (30.7%) CIN3+ cases were identified. Three were invasive cancers, of which two were SCC and one AC; two were AIS and 100 CIN3. Cervical intraepithelial neoplasia grade 2 was diagnosed in another 36 women. The cumulative risk curve of developing CIN3+ after an abnormal cytological test result in our cohort is shown in Figure 2. The 5-year CIN3+ risk was 31.1% (95% CI 26.1–36.6) and the risk in the next 5 years was 1.6% (0.5–4.9). Of all CIN3+, 96.2% (101 out of 105) were detected within 5 years of follow-up.

Table 1 shows the 5- and 10-year risks of developing CIN3+ in 227 hrHPV-positive women (66.4%) and 115 hrHPV-negative women (33.6%). Only 3 (2.9%) of 105 CIN3+ lesions were found in women who were hrHPV negative at baseline. These were all CIN3 lesions. High-risk human papillomavirus-negative women had a 5-year CIN3+ risk of 1.9% (95% CI 0.5–7.0) and a risk of 1.1% (0.2–6.4) in the next 5 years. These risks were 45.1% (95% CI 38.4–52.0) and 2.1% (0.5–7.8), respectively, in hrHPV-positive women. Of the hrHPV-positive women 84.6% (192 out of 227) were infected with a single hrHPV type, 31 (13.7%) had a double infection and four (1.8%) were infected with three or more hrHPV types. All women who developed AIS or invasive cancer had only one hrHPV type: both SCC and one AIS contained HPV16, the AC harboured HPV18, and one AIS was positive for HPV45. In 93.3% (98 out of 105) of CIN3+ cases, the same hrHPV type was present both in the lesion and at the baseline, including both AIS, the AC and one SCC. Of one SCC, no hrHPV typing information was available.

The most prevalent type was HPV16 (105 out of 227, 46.3%), followed by HPV31 (29 out of 227, 12.8%), HPV18 (22 out of 227, 9.7%) and HPV33 (18 out of 227, 7.9%). The CIN3+ risk of women infected with HPV16 was higher than that of women infected with other hrHPV types (Wald-statistic 6.85, *P*=0.009). The 5-year risk in HPV16-positive women was 56.5% (95% CI 46.5–66.0) and this was 36.5% (27.9–46.1) in nonHPV16-positive women. The risks in the subsequent 5 years were 0.01% (95% CI 0.0–10.7) and 3.4% (0.8–12.2), respectively. After stratification in two age categories, we found that in younger women (<30 years), HPV16-positive women had a significantly higher CIN3+ risk than nonHPV16-positive women (Wald statistic 13.01, *P*=0.003, Table 1). Their 5-year CIN3+ risks were 61.5% (95% CI 46.8–74.4) and 19.9% (10.6–34.3), respectively. In older women (⩾30 years), we found no difference in CIN3+ risk between HPV16-positive women and nonHPV16-positive women (Wald statistic 0.08, *P*=0.78). Their respective 5-year risks were 52.1% (95% CI 38.9–65.1) and 51.1% (39.1–63.0).

In women infected with hrHPV types other than HPV16, 18, 31, 33 and/or 45, the CIN3+ risk remained 24.4% (95% CI 14.7–37.7) in the first 5 years and was 2.9% (0.5–15.1) in the following 5 years. These risks were similar for both the age categories.

Women with transient hrHPV infections had a lower CIN3+ risk than women who had a persistent 6-month hrHPV infection (Wald-statistic 17.3; *P*=0.0003). The 5-year CIN3+ risk was 2.2% (95% CI 0.4–12.2) in women who cleared their infection and 56.0% (48.0–63.7) in women with a persistent infection. Within the persistent group, the risk of developing CIN3+ was higher in women positive for HPV16 (67.2%, 95% CI 55.8–76.9) than in women in whom other hrHPV types persisted (45.8%, 35.1–56.9, Wald-statistic 4.73; *P*=0.03).

Women were divided into two groups according to referral cytology; 210 (61.4%) women had a smear of BMD and 132 (38.6%) a smear of >BMD. In both these groups the median of cytological screens between 1996 and 2009 was 3.0 (range 1–9; *P*=0.71, Mann–Whitney).

### Borderline and mild dyskaryosis

**Figure 3:**
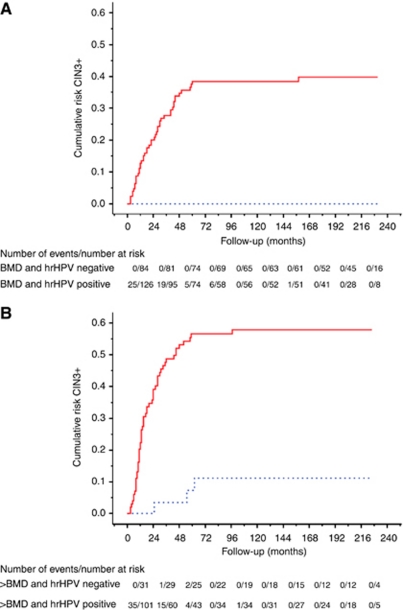
Cumulative risk curve of CIN3+ in women with (**A**) borderline to mild dyskaryosis (*n*=210) and (**B**) moderate to severe dyskaryosis (*n*=132) at baseline, according to baseline hrHPV status. HPV-positive (continuous) women and HPV-negative (dotted) women. Abbreviations: CIN3+, Cervical intraepithelial neoplasia grade 3 or cancer; hrHPV, high-risk human pappillomavirus.

Forty-seven of 210 (22.4%) women with BMD developed CIN3+. Their 5-year CIN3+ risk was 22.5% (95% CI 17.0–29.1) and their risk in the subsequent 5 years was 0.7% (0.1–4.5; Table 2A). Immediate hrHPV testing clearly stratified these women with regard to cumulative risk (Wald-statistic 11.08, *P*=0.001, Figure 3A). A negative hrHPV test result, present in 84 (40.0%) women, reduced the 5-year CIN3+ risk to 0.01% (95% CI 0.0–5.1), whereas a positive test result increased this risk to 37.5% (29.0–46.9). The risks for the subsequent 5 years were 0.01% (95% CI 0.0–5.7) and 1.6% (0.2–9.5%), respectively. Women positive for HPV16 had a higher CIN3+ risk than women infected with other hrHPV types (Wald-statistic 5.60; *P*=0.02). Their 5-year risk was 49.8% (95% CI 36.2–63.4) vs 29.8% (18.4–40.2) in women infected with other hrHPV types. The 5-year risk remained 26.5% (95% CI 14.1–44.3) in women infected with hrHPV types different from HPV16, 18, 31, 33 and 45.

The risk of women who tested hrHPV positive at baseline was further stratified by follow-up testing after 6 months with either cytology or hrHPV (Wald-statistic 8.51; *P*=0.004 and 37.38; *P*<0.0001, respectively).

After women with a follow-up shorter than 6 months had been excluded, the CIN3+ risk of women who complied with the present follow-up algorithm of repeat cytology testing after 6 months was calculated. Women with normal cytology after 6 months had a 5-year CIN3+ risk of 4.9% (95% CI 1.6–13.8), whereas women with an abnormal test result had a risk of 30.9% (23.0–40.1). Risks in the next 5 years were 0.01% (95% CI 0.0–7.1) and 1.4% (0.2–8.5).

After stratification for age, results for both the age groups were statistically not different, although the risks in the younger age group were slightly lower than in the older age group (data not shown).

### >Borderline and mild dyskaryosis

Fifty-eight of 132 (43.9%) women with baseline moderate to severe dyskaryosis developed CIN3+ (Table 2B) and their risk was 45.1% (95% CI 36.4–54.1) in the first 5 years and 3.5% (0.9–12.2) in the subsequent 5 years. Also in this group immediate hrHPV testing stratified the CIN3+ risk (Wald-statistic 12.31, *P*=0.0005, Figure 3B). High-risk human papillomavirus-positive women (76.5%) had a 5-year risk of 56.6% (95% CI 46.4–66.3) and this was 2.9% (0.5–15.4) in the subsequent 5 years. The 5-year risk for women positive for HPV16 was similar to the risk for nonHPV16-positive women (Wald-statistic 1.01; *P*=0.31). Thirty-one (23.5%) women tested hrHPV negative and had a 5-year CIN3+ risk of 7.3% (95% CI 2.0–23.6) and of 4.2% (0.6–23.2) in the subsequent 5 years. Additional testing after 6 months with either cytology, hrHPV, or both did not further stratify the risk (Wald statistic 0.07, *P*=0.80; 0.02, 0.90 and 0.009, 0.93, respectively). Also after age stratification no groups could be identified with a low enough risk to return to routine screening.

### Analyses with CIN2+ as endpoint

Results of analyses with CIN2+ as endpoint were similar to those with CIN3+ as endpoint (Tables 1 and 2). The 5-year CIN2+ risk for women with abnormal cytology was 38.7% (95% CI 33.6–44.1) and this risk was 4.7% (2.4–9.0) between 5 and 10 years. Of all 141 CIN2+ lesions, 124 (87.9%) were detected in women who were hrHPV positive at baseline. Their 5-year CIN2+ risk was 52.3% (95% CI 45.7–58.8) and 6.1% (2.7–13.1) in the next 5 years. Human papillomavirus 16-positive women had a 5-year CIN2+ risk (60.0%, 95% CI 50.2–69.1) similar to the risk in women infected with other hrHPV types (45.7%, 37.0–54.7, Wald-statistic 3.32; *P*=0.07). Women who tested hrHPV negative at baseline had a 5-year risk of 11.8% (95% CI 7.0–19.2) and a risk of 3.3% (1.1–9.6) in the subsequent 5 years.

The 5-year CIN2+ risk in women with BMD was 31.0% (95% CI 25.0–37.8), and their risk in the next 5 years was 3.0% (1.1–7.8). A negative hrHPV-test result at baseline reduced the 5-year risk to 9.9% (95% CI 5.1–18.5), whereas a positive test increased the risk to 44.9% (37.1–52.9). Human papillomavirus 16-positive women had a significantly higher risk than women infected with other hrHPV types (55.3%, 95% CI 41.8–68.1 vs 38.7, 28.1–50.5).

The CIN2+ risk in women with >BMD was 51.2% (95% CI 42.6–59.7) in the first 5 years and 8.4% (3.5–18.8) in the next 5 years. A negative hrHPV test result reduced the 5-year risk to 16.9% (95% CI 7.4–34.2) and a positive hrHPV test result increased this risk to 61.4% (51.5–70.4%). Human papillomavirus 16 had a similar CIN2+ risk as other hrHPV types in women with >BMD (Wald-statistic 0.01; *P*=0.91).

## Discussion

This study describes the long-term cumulative risk of developing CIN3+ after detection of abnormal cytology. For women with an abnormal smear (⩾BMD) the 5-year CIN3+ risk was 31.1% and the risk in the next 5 years was 1.6%. We stratified these risks according to referral cytology and found that both women with BMD and women with >BMD referral cytology had an increased risk of developing CIN3+ within the first 5 years after detection. This risk was twice as high in women with >BMD compared with women with BMD (45% vs 22%). In the subsequent 5 years only for women with >BMD an increased risk (3.5%) remained, while for women referred with BMD this risk was with 0.7% similar to that of the general population (Bulkmans *et al*, 2004).

Immediate hrHPV testing stratified the CIN3+ risk of women with an abnormal smear (⩾BMD). Almost all CIN3+ lesions (102 out of 105), including all invasive carcinomas, were found in women testing hrHPV positive. Almost half of all hrHPV-positive women were infected with HPV16; these women had a significantly higher CIN3+ risk than the women infected with other hrHPV types (Khan *et al*, 2005; Schiffman *et al*, 2005, 2011; Berkhof *et al*, 2006; Castle *et al*, 2009). This risk difference was only found in younger women (<30 years), while in older women (⩾30 years) the risks between women positive for HPV16 and women positive for other hrHPV types were similar. This is in line with another study (Powell *et al*, 2011) that found that the mean age of women with HPV16-associated cancer was significantly lower than of nonHPV16-associated cancer. All CIN3+ in HPV16-positive women were identified within 5 years after detection of abnormal cytology, while lesions associated with other hrHPV types were also found in the 5 years hereafter. This suggests that HPV16 has its main oncogenic effect within a shorter timeframe and at a younger age than other hrHPV types (Safaeian *et al*, 2009; Powell *et al*, 2011).

The CIN3+ risk was lower in women who cleared the virus than in women with persistent hrHPV infections, with the highest risks for women with a persistent HPV16 infection (Schiffman *et al*, 2005; Kjaer *et al*, 2006; Naucler *et al*, 2007; Castle *et al*, 2009).

### Women with BMD

In correspondence with another Dutch study (Berkhof *et al*, 2006), almost 25% of women with BMD developed CIN3+. The majority was diagnosed within the first 5 years, whereas in the subsequent 5-year period their CIN3+ risk was similar to the risk of the general population (Bulkmans *et al*, 2004; Castle *et al*, 2007). This implicates that women with BMD who did not develop CIN3+ within 5 years may return to routine screening. Other studies also found a negligible increase in high-grade CIN cases between 5 and 10 year after diagnosis of BMD cytology (Sherman *et al*, 2003; Khan *et al*, 2005; Schiffman *et al*, 2011).

Meta-analyses concerning women with BMD have found that immediate hrHPV testing better identifies women at risk of developing CIN3+ than repeat cytology after 6 months (Arbyn *et al*, 2004, 2006). Our study confirms these findings. Women with baseline BMD and normal cytology after 6 months (31%) had a 5-year risk of 5%, while this risk was <0.1% in women with BMD and a negative hrHPV test at baseline (40%). Therefore, we support the referral of hrHPV-negative women with BMD to routine screening (Castle *et al*, 2007; Safaeian *et al*, 2007; Rijkaart *et al*, 2009; Katki *et al*, 2011; Levi *et al*, 2011). In both these groups, the 5-year CIN2+ risk remained ∼10%. After revision, all these lesions remained CIN2, and we believe most of them would regress over time. However, additional testing with either cytology or hrHPV after 6 months may be considered to minimise the risk of CIN2.

Women with BMD who tested hrHPV positive at baseline had a 5-year CIN3+ risk of almost 40% and are in need of additional testing and/or colposcopy (Manos *et al*, 1999; Bulkmans *et al*, 2004; Castle *et al*, 2007; Safaeian *et al*, 2007). Although hrHPV genotyping did identify HPV16-positive women to have the highest risk, the risk of women positive for other hrHPV types remained so high (28%) that colposcopic referral was required, leaving hrHPV genotyping without additional value.

Another strategy to identify women at risk for CIN3+ is hrHPV testing after 6 months, allowing viral clearance (Bais *et al*, 2005; Berkhof *et al*, 2005). In our study, almost half (99 out of 210) of the women with BMD tested hrHPV negative after 6 months. As none of them developed CIN3+, this confirms the usefulness of this alternative strategy. However, also with this strategy the 5-year CIN2+ risk in hrHPV-negative women remains 10%.

### Women with >BMD

As almost half of the women with >BMD cytology developed a CIN3+ lesion, we support referring all women with >BMD to colposcopy (Bulkmans *et al*, 2004; Castle *et al*, 2007). Although immediate hrHPV testing did stratify the risk of developing CIN3+, no group was identified with a risk low enough to refrain from colposcopy. Therefore, we do not advocate hrHPV testing in this group.

Our study has several limitations. First, the initial study was designed such that no interference with natural history would occur and therefore had a ‘wait and see’ period to allow the development of real precursor lesions (CIN3), instead of transient lesions (CIN2). When CIN2+ lesions were detected women were treated, which is in contrast to another observational study (McCredie *et al*, 2008). The waiting period is also an explanation for the later diagnosis of CIN3+ lesions in our study than found in a joint European cohort study in which the majority of disease was diagnosed within 12 months (Dillner *et al*, 2008).

Second, our study comprises a relatively small cohort of 342 women. Although the censoring percentage at 10 years was only 20%, just one event was diagnosed after 10 years of follow-up. Therefore, we describe the risks up to 10 years and presented 95% CI to assess all risks as precisely as possible, providing a general impression on the long-term CIN risk of women with an abnormal smear (⩾BMD). Our results corroborate and extend the data of other (long-term) cohorts (Sherman *et al*, 2003; Kjaer *et al*, 2006; Bulk *et al*, 2007; Dillner *et al*, 2008; Katki *et al*, 2011; Kelly *et al*, 2011; Kitchener *et al*, 2011; Schiffman *et al*, 2011). As most CIN3+ were detected within 5 years of referral (Sherman *et al*, 2003), the presented overall 5- and 10-year CIN3+ risks are with 31.1% and 32.2% nearly similar. These risks were higher than reported previously by Dillner (6-year risk 19%) and Sherman (10-year risk 10.2% 95% CI 7.6–12.9) (Sherman *et al*, 2003; Dillner *et al*, 2008). Possible explanations include differences in the study population, that is the relatively large proportion (39%) of women with >BMD, and the strict endpoint of biopsy taking in the initial study. Other studies often acted on less severe suspicions, thereby increasing the detected number of lower grade CIN lesions (Sherman *et al*, 2003; Cuzick *et al*, 2008; Schiffman *et al*, 2011). A number of CIN2 lesions that would have been detected in countries with less conservative referral thresholds, such as the United States and the United Kingdom, will in the Netherlands have developed into CIN3+ lesions before detection. This explains the relatively higher number of CIN3+ lesions and the relatively lower number of CIN2 lesions.

Finally, the median age was relatively low. However, conclusions did not differ greatly after recalculating the risks for 196 women aged ⩾30 years (data not shown).

In conclusion, our study confirms the increased CIN3+ risk in women with dyskaryosis. Bearing in mind the limitations of our study, we recommend the following:

Women with BMD should receive additional hrHPV testing for risk assessment. HrHPV-negative women may be referred to routine screening as their 5-year CIN3+ risk is negligible. HrHPV-positive women should be referred for additional testing and/or colposcopy. When these women do not develop CIN3+ within 5 years, they also may be referred to population-based screening. Women with >BMD should all be referred for colposcopy and as their CIN3+ risk is elevated for at least 10 years long-term monitoring is required.

## Figures and Tables

**Table 1:** Value of hrHPV testing during the follow-up of women with abnormal baseline cytology; 5-year and 10-year risks

		**CIN3+**				**CIN2+**			
		**Risk**	**5 Year**	**Risk**	**10 Year**	**Risk**	**5 Year**	**Risk**	**10 Year**
**Baseline**	**At risk**	**(%)**	**95% CI**	**(%)**	**95% CI**	**(%)**	**95% CI**	**(%)**	**95% CI**
All women	342	31.1	26.1–36.6	32.2	26.9–38.0	38.7	33.6–44.1	41.6	36.2–47.2
HPV negative	115	1.9	0.5–7.0	3.0	0.9–9.2	11.8	7.0–19.2	14.7	9.0–23.1

*HPV positive*	227	45.1	38.4–52.0	47.0	39.9–54.2	52.3	45.7–58.8	55.2	48.4–61.8
HPV16	105	56.5	46.5–66.0	56.5	46.1–66.3	60.0	50.2–69.1	61.1	51.0–70.3
NonHPV16	122	36.5	27.9–46.1	38.7	29.5–48.8	45.7	37.0–54.7	50.2	41.0–59.3
NonHPV16, 18, 31, 33, 45	61	24.4	14.7–37.7	26.6	16.1–40.6	36.4	25.3–49.2	41.8	29.9–54.7
									
*Age < 30 years*									
HPV positive	104	40.4	30.7–51.0	41.8	31.6–52.8	49.5	39.8–59.3	54.0	43.9–63.8
HPV16	49	61.5	46.8–74.4	61.5	46.2–74.8	65.8	51.4–77.8	58.1	43.5–71.4
NonHPV16	55	19.9	10.6–34.3	22.8	12.3–38.3	35.2	23.6–48.9	41.5	28.8–55.4

*Age ⩾30 years*									
HPV positive	123	51.5	42.5–60.4	51.5	42.1–60.8	55.5	46.6–64.1	56.4	47.2–65.2
HPV16	56	52.1	38.9–65.1	52.1	38.3–65.5	55.2	42.0–67.7	55.2	41.5–68.1
NonHPV16	67	51.1	39.1–63.0	51.1	38.5–63.6	55.7	43.8–67.0	57.3	44.9–68.8

Clearance <6 months^a^	50	2.2	0.4–12.2	2.2	0.3–13.1	14.6	7.2–27.3	14.6	7.0–28.0

*6-Month persistence* a	166	56.0	45.0–63.7	57.5	49.2–65.4	61.0	53.2–68.2	64.4	56.5–71.6
Persistence HPV16	77	67.2	55.8–76.9	67.2	55.4–77.2	68.4	57.1–77.9	69.9	58.4–79.4
Persistence nonHPV16	89	45.8	35.1–56.9	48.9	37.6–60.4	54.7	44.1–64.9	59.8	48.9–69.8

Abbreviations: CIN3+=cervical intraepithelial neoplasia grade 3 and cancer; CIN2+=cervical intraepithelial neoplasia grade 2, 3 and cancer; 95% CI=95% confidence interval; hrHPV=high-risk human papillomavirus.

a All hrHPV-negative women at baseline and all women with a follow-up of <6 months were excluded.

6-Month persistence: at baseline and at 6 months at least one detected hrHPV type is similar. Time to event is set equal to histological diagnosis of CIN3+ or CIN2+ lesion.

**Table 2:** Risk (%) of cytology and hrHPV testing at baseline and at 6-month follow-up, stratified according to baseline cytology in BMD and >BMD

**(A) Women with borderline and mild dyskaryosis**										
			**CIN3+**				**CIN2+**			
		**BMD**	**Risk**	**5 Year**	**Risk**	**10 Year**	**Risk**	**5 Year**	**Risk**	**10 Year**
**Baseline**	**Follow-up (6 months)^a^**	**At risk**	**(%)**	**95%CI**	**(%)**	**95%CI**	**(%)**	**95%CI**	**(%)**	**95%CI**
All		210	22.5	17.0–29.1	23.1	17.4–30.4	31.0	25.0–37.8	33.0	26.6–40.1
	Cytology negativeb,c	65	4.9	1.6–13.8	4.9	1.6–14.3	12.5	6.4–22.9	12.5	6.3–23.4
	Cytology positiveb,d	127	30.9	23.0–40.1	31.8	23.3–41.7	38.0	29.7–47.0	41.5	32.7–50.9
										
HPV negative		84	0.0	0.0–5.1	0.0	0.0–5.7	9.9	5.1–18.5	11.2	5.8–20.5
	Cytology negative^b^	37	0.0	0.0–10.2	0.0	0.0–10.7	5.5	1.5–18.1	5.5	1.5–18.6
	Cytology positive^b^	40	0.0	0.0–10.7	0.0	0.0–12.5	10.9	4.3–25.1	13.7	5.7–29.5
	HPV negative	70	0.0	0.0–6.1	0.0	0.0–6.6	10.4	5.1–20.1	10.4	5.1–20.5
	HPV positive	9	0.0	0.0–35.4	0.0	0.0–43.4	12.5	2.2–47.1	25.0	6.6–61.1
	Double negative	36	0.0	0.0–10.4	0.0	0.0–11.0	5.6	1.5–18.5	5.6	1.5–19.0
	Cytology and/or HPV positive	48	0.0	0.0–9.2	0.0	0.0–10.4	13.4	6.2–26.4	15.7	7.5–30.1
										
HPV positivec,d		126	37.5	29.0–46.9	38.5	29.5–48.4	44.9	37.1–52.9	47.6	38.5–56.9
	Cytology negative^b^	28	11.6	3.9–29.9	11.6	3.6–31.5	22.0	10.5–40.5	22.0	10.0–41.7
	Cytology positivec,d	87	44.9	34.2–56.1	46.3	35.0–57.9	50.3	39.6–60.9	54.2	43.1–64.9
	HPV negative	29	0.0	0.0–13.8	0.0	0.0–15.5	11.0	3.8–27.9	11.0	3.6–29.2
	HPV positivec,d	91	46.3	35.8–57.1	47.6	36.5–59.0	52.2	41.8–62.4	55.9	45.0–66.3
	Double negative	15	0.0	0.0–22.8	0.0	0.0–25.9	6.7	1.1–30.9	6.7	1.0–33.4
	Cytology and/or HPV positive^c^,d	105	40.6	31.1–50.9	41.2	31.1–52.0	47.4	37.8–57.1	50.6	40.6–60.6
HPV16		55	49.8	36.2–63.4	49.8	35.6–64.0	55.3	41.8–68.1	57.4	43.4–70.3
nonHPV16		71	29.8	18.4–40.2	29.8	19.3–43.0	38.7	28.1–50.5	40.3	29.0–52.7
										
**(B) Women with moderate and severe dyskaryosis**										
			**CIN3+**				**CIN2+**			
		**>BMD**	**Risk**	**5 Year**	**Risk**	**10 Year**	**Risk**	**5 Year**	**Risk**	**10 Year**
**Baseline**	**Follow-up (6 months)^a^**	**At risk**	**(%)**	**95%CI**	**(%)**	**95%CI**	**(%)**	**95%CI**	**(%)**	**95%CI**
All		132	45.1	36.4–54.1	47.0	37.8–56.4	51.2	42.6–59.7	55.3	46.4–63.9
	Cytology negative^b^	21	5.0	0.9–23.6	15.0	5.1–36.7	5.0	0.9–23.6	15.0	5.1–36.7
	Cytology positive^b^	98	53.9	43.5–64.0	53.9	42.9–64.5	60.7	50.7–69.9	64.0	53.7–73.2
										
HPV negative		31	7.3	2.0–23.6	11.2	3.4–31.0	16.9	7.4–34.2	24.2	11.6–43.6
	Cytology negative^b^	10	0.0	0.0–29.9	11.1	1.8–45.6	0.0	0.0–29.9	11.1	1.8–45.6
	Cytology positive^b^	18	13.5	3.7–39.1	13.5	3.0–44.3	29.4	13.3–53.1	36.5	16.9–61.9
	HPV negative	30	7.4	2.0–23.8	11.3	3.5–31.1	14.1	5.6–31.3	21.6	9.7–41.3
	HPV positive	1	0.0	-	0.0	-	100	20.7–100	100	20.7–100
	Double negative	10	0.0	0.0–29.9	11.1	1.8–45.6	0.0	0.0–29.9	11.1	1.8–45.6
	Cytology and/or HPV positive	21	11.2	3.0–33.7	11.2	2.6–37.5	25.0	11.2–46.9	30.8	14.2–54.5
										
HPV positive^e^		101	56.6	46.4–66.3	57.8	47.2–67.7	61.4	51.5–70.4	64.6	54.6–73.5
	Cytology negative^b^	11	9.1	1.6–37.8	18.2	5.1–47.7	9.1	1.6–37.8	18.2	5.1–47.7
	Cytology positiveb,e	82	62.2	50.7–72.4	62.2	50.3–72.8	67.3	56.4–76.6	69.1	58.0–78.4
	HPV negative	14	0.0	0.0–24.3	0.0	0.0–25.9	7.7	1.4–33.3	7.7	1.3–34.6
	HPV positive^e^	81	65.1	53.7–75.0	66.8	55.1–76.7	68.8	57.9–77.9	72.8	62.0–81.5
	Double negative	5	0.0	0.0–43.4	0.0	0.0–43.4	0.0	0.0–43.4	0.0	0.0–43.4
	Cytology and/or HPV positive^e^	91	58.0	47.2–68.1	59.4	48.2–69.7	62.7	52.3–72.1	66.3	55.7–75.4
HPV16		50	63.7	49.6–75.8	63.7	49.1–76.2	65.3	51.3–77.1	65.3	50.8–77.4
NonHPV16		51	48.7	34.5–63.1	51.5	36.7–66.0	57.5	43.6–70.3	63.8	49.8–75.8

Abbreviations: CIN3+=cervical intraepithelial neoplasia grade 3 and cancer; CIN2+=cervical intraepithelial neoplasia grade 2, 3 and cancer; 95% CI=95% confidence interval; HPV=human papillomavirus.

a All women with a follow-up of <6 months were excluded.

b Cytology divided into negative (normal) and positive (borderline or mild dyskaryosis and worse).

c Including one adenocarcinoma *in situ* (AIS).

d Including one squamous cell carcinoma (SCC).

e Including one SCC, one adenocarcinoma, and one AIS.

Time to event is set equal to histological diagnosis of CIN3+ lesion.
